# Enhancing Smart Building Surveillance Systems in Thin Walls: An Efficient Barrier Design

**DOI:** 10.3390/s24020595

**Published:** 2024-01-17

**Authors:** Taewoo Lee, Hyunbum Kim

**Affiliations:** Department of Embedded Systems Engineering, Incheon National University, Incheon 22012, Republic of Korea; hst0092@inu.ac.kr

**Keywords:** smart building, surveillance, infrastructure, walls, efficiency

## Abstract

This paper introduces an efficient barrier model for enhancing smart building surveillance in harsh environment with thin walls and structures. After the main research problem of minimizing the total number of wall-recognition surveillance barriers, we propose two distinct algorithms, Centralized Node Deployment and Adaptation Node Deployment, which are designed to address the challenge by strategic placement of surveillance nodes within the smart building. The Centralized Node Deployment aligns nodes along the thin walls, ensuring consistent communication coverage and effectively countering potential disruptions. Conversely, the Adaptation Node Deployment begins with random node placement, which adapts over time to ensure efficient communication across the building. The novelty of this work is in designing a novel barrier system to achieve energy efficiency and reinforced surveillance in a thin-wall environment. Instead of a real environment, we use an ad hoc server for simulations with various scenarios and parameters. Then, two different algorithms are executed through those simulation environments and settings. Also, with detailed discussions, we provide the performance analysis, which shows that both algorithms deliver similar performance metrics over extended periods, indicating their suitability for long-term operation in smart infrastructure.

## 1. Introduction

In the era of smart cities, the concept of smart buildings has emerged as a vital component of urban infrastructure to provide convenient lives to citizens. In the realm of smart buildings, seamless communication and continuous surveillance between various components of the infrastructure are crucial. One of the primary obstacles in establishing such communication is the presence of physical barriers like walls—structures which can significantly disrupt data flow, diminish detection accuracy, and affect the system’s overall performance. These buildings are characterized by their integration of IoT (Internet of Things) technologies, facilitating advanced automation and real-time control over various subsystems, such as energy management, security, and environmental controls [1,2,3,4,5,6,7,8]. Also, it is highly anticipated that 5G and 6G communication technologies are utilized in smart buildings and expansive spaces [9,10,11,12,13,14,15].

However, the increase in smart buildings and their effective operations depend on the availability and efficiency of communication systems in these structures. One of the most significant challenges faced in deploying effective communication systems within smart buildings is the presence of physical barriers, primarily walls, which can significantly disrupt the flow of data. These result in coverage gaps and reductions in the overall performance of the communication system. This issue becomes exacerbated when we deliberate on solid walls, which present a formidable obstacle to the propagation of electromagnetic waves, leading to disconnected spaces within the building and jeopardizing the optimal functioning of the smart system.

On the other hand, surveillance and secure monitoring are considered as critical tasks in smart buildings; these tasks can be supported by heterogeneous mobile robots, drones, UAVs (Unmanned Aerial Vehicles), IoT devices, and intelligent components [16,17,18,19,20,21,22]. To provide reinforced surveillance and secure monitoring, the concept of a barrier can be applied to smart buildings because the formation of a barrier and its creation guarantee that any penetrations or mobile objects are detected by system members in the built surveillance barriers within the requested three-dimensional space and plane area [23,24,25,26,27,28,29]. Essentially, it has been known that the barrier has been used for numerous applications, including virtual emotion surveillance, digital twins, maritime transportation systems, public and private areas, patrol services, border surveillance, smart complex surveillance, virtual emotion informatics, and geographic segmentation surveillance [30,31,32,33,34]. However, it is not sufficient to form secure building surveillance in smart buildings with the walls in order to deliberate on energy-efficient formations when the walls are present because these may affect the detection and the communication by the deployed system members or components equipped with wireless transmitters and receivers. Thus, it is highly necessary to proceed by handling the issue so as to enhance secure surveillance in smart buildings with consideration of energy efficiency.

To solve this issue, this paper proposes an approach: the preemptive placement of communication nodes within thin walls. The idea is to leverage the thin walls, seen as obstacles, as conduits for communication. By strategically embedding nodes within these walls and positioning additional nodes in the adjacent spaces, a communication barrier can be created. This barrier bridges the disconnect caused by the physical walls, thereby enabling seamless data transfer between spaces.

The objective function of this study is to pursue energy-efficient surveillance in smart buildings within the walls. It follows that the main task in the proposed system is to minimize the number of nodes or system members while ensuring optimal communication with surveillance. This consideration is important, as an excessive number of nodes can lead to higher costs, increased energy consumption, and potential signal interference. The potential for signal interference also rises with an increase in the number of nodes. As more nodes are placed in proximity, there is a higher likelihood of overlapping signals, which can lead to data corruption and a drop in network performance [8]. Therefore, the balance between the number of nodes and communication effectiveness is a complex challenge that requires a careful and innovative approach to ensure that smart buildings can function efficiently without incurring prohibitive costs.

Based on the above motivations, the main contributions of the paper are summarized below:

- First, we design an efficient barrier system for enhancing smart building surveillance in harsh environments with walls and infrastructure. The proposed system is designed to consider energy-efficient surveillance and optimal formations of system components;
- Then, this paper presents a formal definition of the research problem to minimize the number of nodes or system components, in order to ensure secure surveillance and communication among system components;
- To resolve the problem, we propose two different algorithms for the preemptive placement of nodes within thin walls and the adjacent spaces. These algorithms aim to minimize the number of nodes to an optimal level and to optimize their placement, striking a balance between system efficiency, cost effectiveness, and environmental sustainability;
- Instead of real circumstances, we utilize an ad hoc server for simulations with various scenarios and parameters. Then, the performances of the proposed algorithms are analyzed for obtained outcomes through those simulations using various settings and scenarios; as well, detailed discussions are provided for the obtained results.

In the following sections of this paper, we have systematically arranged our discussion and analysis to offer a comprehensive perspective on our research. In Section 2, we provide a detailed problem definition and present an overview of the system. This section serves as the foundation of our study, outlining the specific challenges associated with communication in smart buildings and the system parameters that our proposed algorithms operate within. In Section 3, we introduce our two algorithms for the preemptive placement of nodes within thin walls and the adjacent spaces. Each algorithm is explained in detail, including its design principles, operation, and expected performance characteristics. Our objective here is to provide a thorough understanding of the mechanisms of these algorithms and how they aim to solve the problem defined in Section 2. In Section 4, we delve into an evaluation of our proposed algorithms. Using a series of simulations, we illustrate the performance of each algorithm under various conditions. This section provides explanations of how our algorithms function, demonstrating their potential to improve communication within smart buildings. In Section 5, we conduct a comparative study of the two algorithms. Drawing on the results from the previous section, we analyze and contrast the performance of each algorithm. This comparative analysis allows us to identify the relative strengths and weaknesses of each algorithm, offering valuable insights into which one provides a more optimal solution to the problem of communication disruption and secure surveillance with energy efficiency in smart buildings.

## 2. Proposed Framework

First of all, we design the efficient barrier model for solidifying smart building surveillance in harsh environments with walls and structures. And the essential terms and definitions in regard to the proposed model are represented. Also, the primary research problem in the paper is formally defined.

### 2.1. System Overview and Assumptions

The proposed system revolves around a smart building environment, considered as a three-dimensional space, wherein certain areas are obstructed by thin walls that act as physical barriers for communication. These walls divide the space into two parts, creating a challenge for data transfer between different sections of the building. The system members, or sensors, are randomly deployed throughout the available space, excluding the wall. These sensors are the key components in our communication system, serving as the nodes that facilitate data transfer across the building. Their placement is random, reflecting the unpredictability and variation found in real-world deployment. The wall in this system, though physically thin, is considered impenetrable for the communication signals used by the sensors.

**Figure 1:**
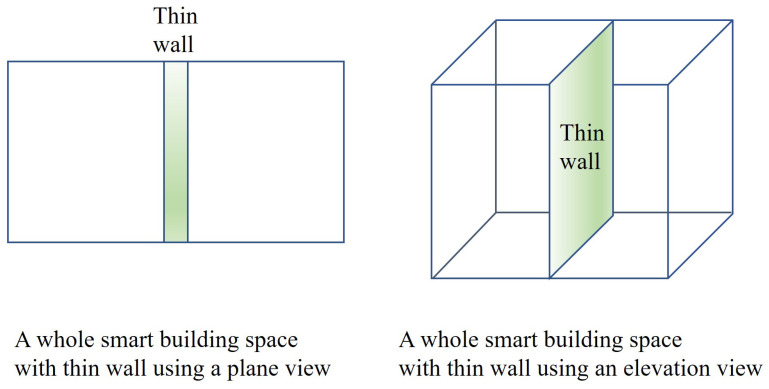
A brief overview of the whole space.

Figure 1 depicts a brief overview of the given space. When we consider a two-dimensional plane, a thin wall is located vertically, which may affect surveillance and communication between the left border and right border.

Then, the below assumptions and settings are engraved to activate the proposed system:

- The three-dimensional space is considered as the region of interest within the smart building as a whole. And the smart building contains thin walls, which are located everywhere within the building;
- The proposed system consists of a group of system members or components, including IoT devices, mobile robots, and sensors, where each component has equal detection or communication range and is equipped with wireless transmitters and receivers;
- The connection between two system members is created if there exists an overlapped space between the detection ranges of two neighbors.

### 2.2. Notations, Essential Terms, Problem Definition

The basic terms which are utilized in the proposed system are presented and the main research problem is also defined in this subsection. The goal is to create a barrier in a three-dimensional space, reducing the number of nodes. There is a very thin wall in the space that separates the two spaces. The input is the sensing radius and the output returns the number of nodes used when the number of barriers is greater than or equal to a certain number of barriers.

**Definition** **1** *(wall-recognition surveillance security barriers)**.** Suppose that there is a smart building space, where the space includes walls or similar complex infrastructure that may affect wireless communication, data transfer, transmission, and reflection. Given the space with thin walls, the system allows heterogeneous members, including a group of IoT devices, mobile robots, sensors, and cameras, which are equipped with a wireless transmitter and receiver. And each member has the maximum allowed number of connections through the thin wall that covers one hop distance or detection range of the system member. The wall-recognition surveillance security barriers in smart buildings, called WalRecogSurv, are constructed by a sub-group of system members to detect any penetration or object movement between specific directions.*

**Definition** **2** *(MinWalRecogSurv problem)**.** It is given that it is necessary to generate a group of wall-recognition surveillance security barriers in a smart building. The MinWalRecogSurv problem is to minimize the total number of wall-recognition surveillance security barriers in the smart building environment, such that the requested allowed number of connections through walls or installations within the walls is satisfied.*

Hence, the objective of the *MinWalRecogSurv* problem is to

(1) Minimizeδ

Also, the indispensable notations, with their brief descriptions and explanations, are summarized in Table 1.

## 3. Proposed Methods

This section presents our proposed schemes to resolve the *MinWalRecogSurv* problem in the smart building space. The implementation processes and steps of both approaches are specified.

### 3.1. Algorithm 1: Centralized Node Deployment

First of all, an approach for centralizing node position, referred to as *Centralized Node Deployment*, is devised to solve the *MinWalRecogSurv* problem, which returns δ as the minimal number of system members required to build wall-recognition surveillance security barriers. The *Centralized Node Deployment* scheme largely consists of the following steps:

- The first step is to place the nodes in a row along the centerline of the thin wall. This centralized deployment ensures that nodes are evenly distributed along the length of the wall, which is important for maintaining consistent communication coverage;
- When nodes are placed inside thin walls, the algorithm randomly deploys nodes on both sides of adjacent walls. Randomness here means that nodes are placed at various points on adjacent walls, but within a defined range, to ensure effective signal transmission with nodes within thin walls. This step introduces a variation factor that reflects real-world conditions, in which nodes can be placed in various locations, depending on the specific requirements and constraints of the building;
- The final step is to form a communication barrier based on nodes placed inside the thin wall by [35]. This barrier overcomes communication interruptions caused by thin walls and enables effective data transfer between randomly placed nodes on either side of the wall. The formation of this communication barrier optimizes communication paths between nodes and improves data transmission within smart buildings. Then, we estimate the total number of current surveillance barriers and return it as the final outcome.

**Figure 2:**
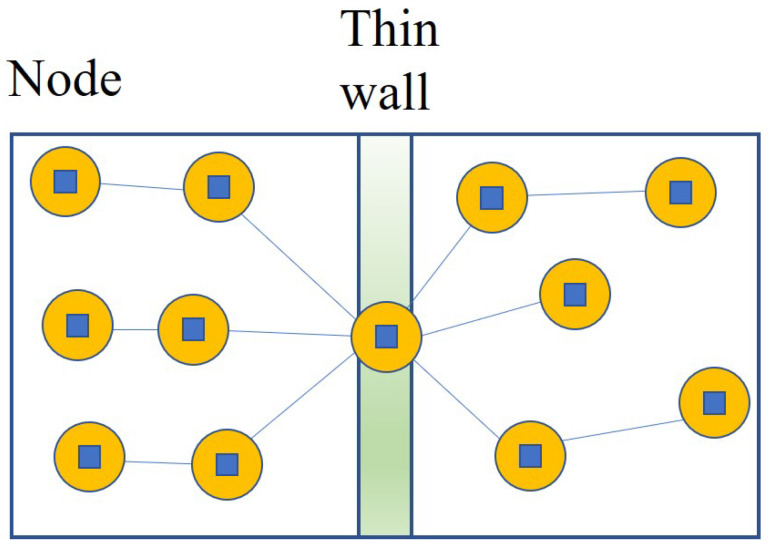
The implementation procedure of Algorithm 1 along the centerline of the wall.

**Figure 3:**
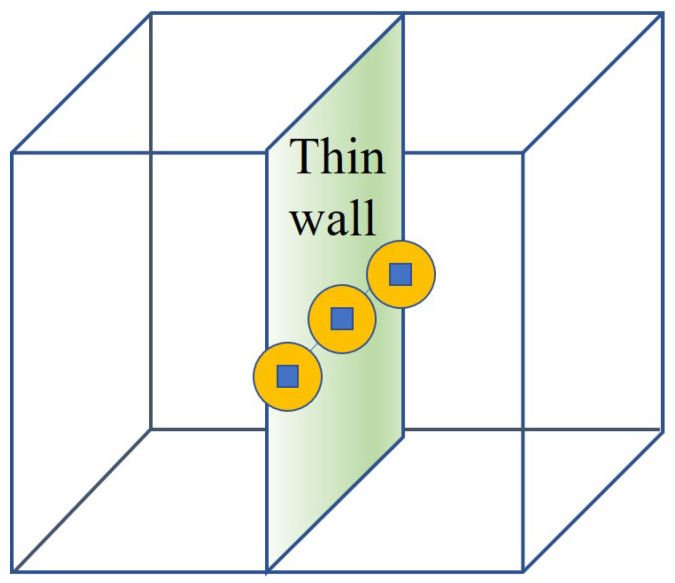
The execution status of Algorithm 1 with the determined node deployments within the wall.

Figure 2 shows the implementation strategy of Algorithm 1: *Centralized Node Deployment*, with consideration of a centerline in the wall. As can be seen in Figure 2, such a strategy ensures that system members or nodes are distributed evenly in the given smart building space. Also, Figure 3 depicts the executed state of Algorithm 1: *Centralized Node Deployment*. As shown in Figure 3, Algorithm 1 helps the fair distribution of system members through the wall when the wall-recognition surveillance security barriers in the smart building space are created with the requested number of allowed connections through the wall, or installations within the wall, consequently.
**Algorithm 1** Centralized Node Deployment Inputs: *S*, *M*, *r*, *t*, *q*, Output: *δ*
  1:  verify *M* with *r* within *S*;
  2:  recognize the walls in *S*;
  3:  set *W* ← ∅;
  4:  place nodes in centerline in the walls;
  5:  **while** *q* number of *WalRecogSurv* are not formed **do**
  6:         seek a new *WalRecogSurv* through the centerline in the walls with *t* and *p*;
  7:         **if** a new *WalRecogSurv w_k_* is found **then**
  8:               set $W\leftarrow W\cup w_{k}$;
  9:         **end if**
10:  **end while**
11:  calculate |W|;
12:  update |W| to *δ*;
13:  return *δ*;


Furthermore, the pseudocode of *Centralized Node Deployment* is explained in Algorithm 1 with formal representations.

### 3.2. Algorithm 2: Adaptation Node Deployment

Secondly, an approach for adapting node position, called *Adaptation Node Deployment*, is developed to work out the defined *MinWalRecogSurv* problem, seeking the minimal number of system members such that the requested number of allowed connections through the wall, or installations within the wall, is met. Then, the *Adaptation Node Deployment* approach is largely composed of the procedures below:

- The first step assumes that there is no wall and randomly deploys nodes in the entire space of the smart building. This random placement reflects the variability in and irregularity of node placement in the real world;
- This step creates a barrier based on the initial node placement by [35]. This barrier assumes that there is no wall and forms a communication flow between nodes; each node can transmit and receive data to and from adjacent nodes. After the barrier is created, it finds this flow to see how communication is formed;
- After finding the flow, it finds the point where the flow and the wall intersect. This intersection is an area where communication disconnection may occur, and additional nodes are placed at that point to resolve this. This keeps the communication flow through the wall smooth and enables data transfer to other areas within the smart building. Then, we measure the total number of current surveillance barriers and return it as final result.

**Figure 4:**
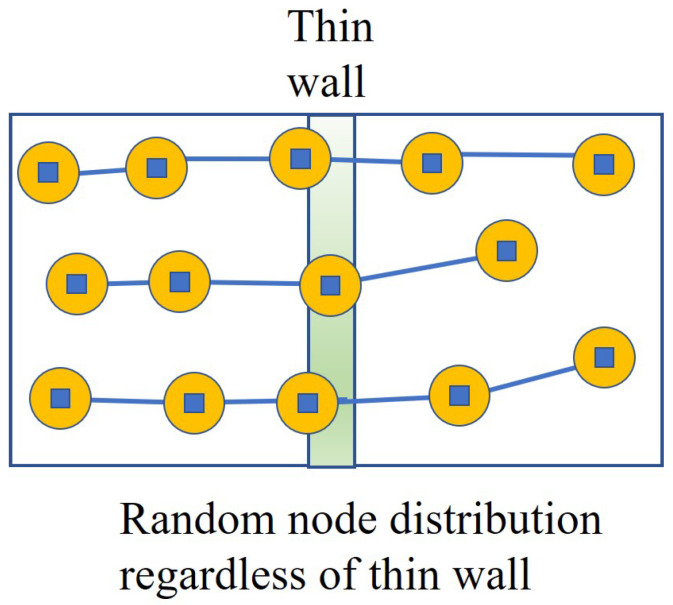
The arbitrary deployment of Algorithm 2.

**Figure 5:**
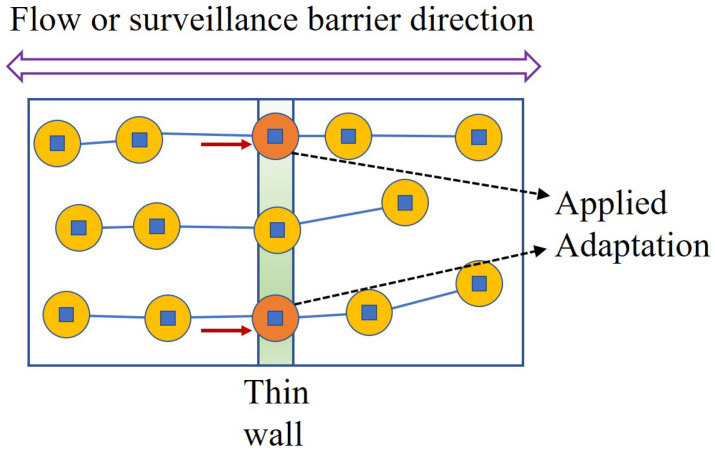
The implementation procedure of Algorithm 2 with consideration of adapted flows.

**Figure 6:**
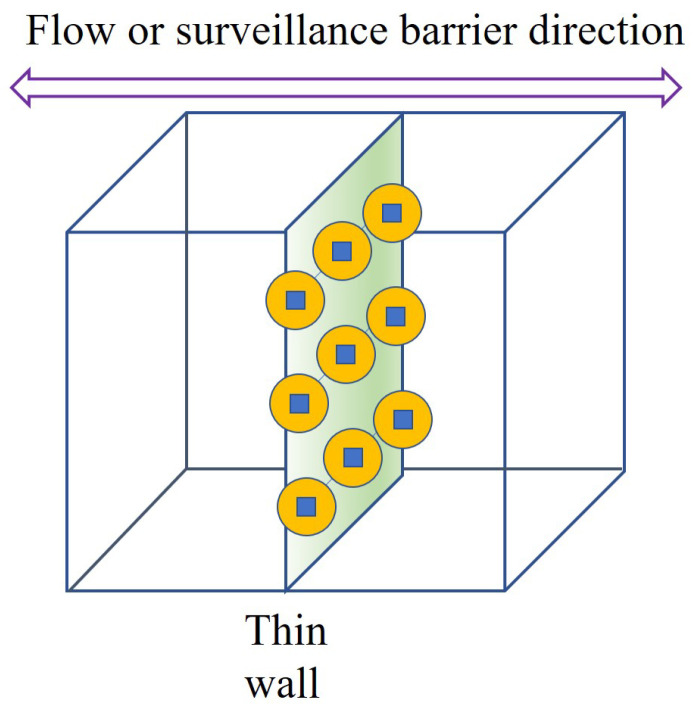
The execution status of Algorithm 2 with the node locations adopted within the wall.

Figure 4 presents the arbitrary deployment of Algorithm 2: *Adaptation Node Deployment*. The random deployments are performed through the entire space of the smart building. Then, Figure 5 describes the implementation procedure of Algorithm 2. As can be seen in Figure 5, Algorithm 2 searches for the flow to see how communication is formed after the barriers are generated. Moreover, Figure 6 shows the execution status of Algorithm 2 with the node locations adopted within the wall. It follows that, after finding the flows, Algorithm 2 recognizes the wall intersections so that it keeps the communication flow and the detection through the wall within the smart building.
**Algorithm 2** Adaptation Node Deployment
Inputs: *S*, *M*, *r*, *t*, *q*, Output: *δ*

  1:  identify *M* with *r* within *S*;
  2:  detect the walls in *S*;
  3:  set *W* ← ∅;
  4:  **while** *q* number of flows are not generated **do**
  5:      seek a new flow between left border and right border with *t* and *p*;
  6:      **if** a new flow is found **then**
  7:            add it to *W*;
  8:      **end if**
  9:  **end while**
10:  calculate |W|;
11:  search for the points where the flow and the wall intersect;
12:  add those points to |W|;
13:  update |W| to *δ*;
14:  return *δ*;


Moreover, the pseudocode of *Adaptation Node Deployment* is specified in Algorithm 2, based on formal notations and descriptions.

## 4. Performance Analysis

In this section, we evaluate the performances of the proposed Algorithm 1: *Centralized Node Deployment* and Algorithm 2: *Adaptation Node Deployment* after several groups of simulations are performed. In the simulations, we utilize several settings and parameters, covering different sensing or detection ranges of system members, different numbers of connections through the wall, different possible numbers of connections among system members, several numbers of wall-recognition surveillance security barriers, etc. The simulation settings are summarized as follows. The size of the smart building is considered as a 1000 (width) × 1000 (height) × 1000 (depth) space. The sensing or detection range of system member *r* ranges from 50 to 200, where each system member has equal detection radius. In essence, a sole thin wall in the smart building is considered in each simulation. The allowed number of connections through the wall *t* ranges from 10 to 30. And the possible number of connections among system members *p* is considered between 1 and 4. Also, the requested number of wall-recognition surveillance security barriers ranges from 20 through 50. As such, the objective value of δ is the number of system members, which is the final output value of the proposed algorithms and the average value of 100 different graphs and experiments. All experiments are conducted using $C^{++}$ in an arm64cpu computer; the resulting graphs are created by MATLAB.

First, our schemes are described in Table 2, including the pros and cons compared to other studies.

**Figure 7:**
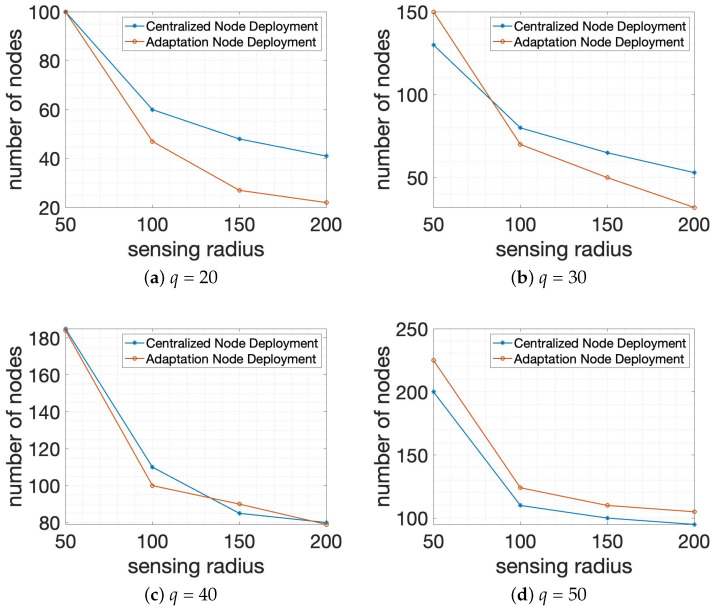
Performance comparison for the total number of nodes or system members of the requested number of wall-recognition surveillance security barriers *q* in different sensing ranges with the allowed number of connections through the wall *t* = 20 and *p* = 3 in 1000×1000×1000 smart building size.

In the first group of experiments, Algorithm 1: *Centralized Node Deployment* and Algorithm 2: *Adaptation Node Deployment* are performed over different sensing ranges with the allowed number of connections through the wall *t* = 20 and *p* = 3 in the 1000×1000×1000 smart building size, as shown in Figure 7. It is noted that the experimental outcome is composed of two axes, so that the X-coordinate specifies the sensing range of the system members and the Y-coordinate presents the total number of system members δ of objective value, so as to build the requested number of wall-recognition surveillance security barriers completely. In Figure 7, sensing radius or detection range has been set as 50, 100, 150, or 200. Figure 7a,b demonstrates the performance of two different algorithms according to different sensing ranges with *q* = 20 and *q* = 30, respectively. Also, Figure 7c,d shows the performance comparison of two algorithms when *q* = 40 and *q* = 50 are given in the experiment. As shown in Figure 7, it is verified that the total number of system members δ is decreasing as the sensing range of node is increasing because the bigger sensing range allows more space to be detected by each node. Also, we can confirm that Algorithm 2: *Adaptation Node Deployment* shows better performance than Algorithm 1: *Centralized Node Deployment* in the first experimental scenario.

**Figure 8:**
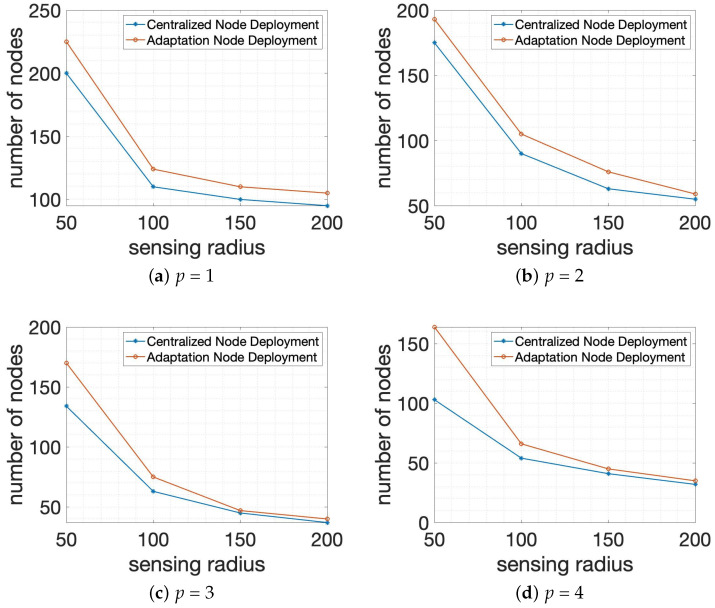
Performance comparison for the total number of nodes or system members of the possible number of connections among system members in different sensing ranges with the allowed number of connections through the wall *t* = 20 and *q* = 50 in 1000×1000×1000 smart building size.

For the second set of simulations, we also executed two algorithms, Algorithm 1: *Centralized Node Deployment* and Algorithm 2: *Adaptation Node Deployment*, using various sensing radii with the allowed number of connections through the wall *t* = 20 and *q* = 50 in 1000×1000×1000 smart building size, as can be seen in Figure 8. Similar to the first group of experiments, the experimental outcome results consist of two axes, where the X-coordinate represents the sensing radius of system members and the Y-coordinate specifies the total number of system members δ as the final outcome. In Figure 8, sensing radius or detection range has been set as 50, 100, 150, or 200. Figure 8a,b shows the performance comparison if two algorithms are executed with *p* = 1 and *p* = 2. And Figure 8c,d stands for the performance of two algorithms when *p* = 3 and *p* = 4 are utilized in the system. According to Figure 8, it is observed that the total number of system members δ is decreasing significantly as the sensing range of the node is increasing. The reason is that the larger sensing range gives more opportunity to search for neighbors or system members when the wall-recognition surveillance security barriers are formed. Moreover, it is demonstrated that Algorithm 2: *Adaptation Node Deployment* outperforms Algorithm 1: *Centralized Node Deployment* for all applicable missions in the second scenario of simulation. And the performance difference between two algorithms is diminished if the sensing range of system members increases.

**Figure 9:**
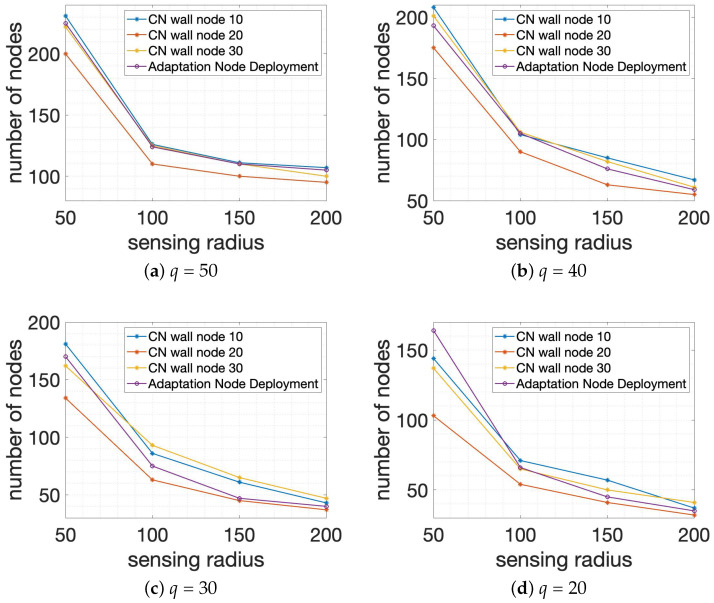
Performance comparison for the total number of nodes or system members of the requested number of wall-recognition surveillance security barriers *q* in different sensing ranges with the allowed number of connections through the wall *t* = 10, 20, 30 and *p* = 3 in 1000×1000×1000 smart building size.

Lastly, as the third group of experiments, we achieved two schemes for Algorithm 1: *Centralized Node Deployment* and Algorithm 2: *Adaptation Node Deployment* based on the scenario that covers the requested number of wall-recognition surveillance security barriers *q* in different sensing ranges with the allowed number of connections through the wall *t* = 10, 20, 30 and *p* = 3 in 1000×1000×1000 smart building size. In particular, Algorithm 1: *Centralized Node Deployment* with various *t* values for the allowed number of connections through the wall is implemented and is compared with Algorithm 2: *Adaptation Node Deployment*. Similar to previous groups of experiments, the simulation results are presented with two axes, in which the X-coordinate stands for the sensing radius of system members and the Y-coordinate represents the total number of system members δ for the obtained result. Figure 9a,b depicts the performance comparison for Algorithm 1: *Centralized Node Deployment* with the allowed number of connections through the wall *t* = 10, 20, 30 and Algorithm 2: *Adaptation Node Deployment*, depending on *q* = 50 and *q* = 40. And Figure 9c,d presents the results of Algorithm 1: *Centralized Node Deployment* with *t* = 10, 20, 30 and Algorithm 2: *Adaptation Node Deployment* when the requested number of wall-recognition surveillance security barriers *q* = 30 and *q* = 20 are given. From Figure 9, it is identified that the total number of system members δ is decreasing significantly as the sensing range of the node is increasing as a whole because the larger sensing range of each node gives a higher chance to cover a wide space and to connect with other nodes. In addition, Algorithm 1: *Centralized Node Deployment* with *t* = 20 has the best performance compared to the other cases in the third scenario of simulation.

## 5. Conclusions

In this paper, we proposed and evaluated two distinct algorithms, *Centralized Node Deployment* and *Adaptation Node Deployment*, to overcome the challenge of communication disruption in smart buildings caused by physical barriers like thin walls. Our findings underscore the effectiveness of both algorithms, with their unique deployment strategies contributing to the optimal functioning of the communication system within the building. The *Centralized Node Deployment* algorithm, with its strategic node placement along the thin walls, proved effective in maintaining consistent communication coverage and effectively mitigating potential communication disruptions. Notably, it showed superior performance as the number of required barriers increased, indicating its ability to handle complex communication obstacles. On the other hand, the *Adaptation Node Deployment* algorithm, starting with random node placement and adapting over time, also demonstrated its capability to ensure efficient communication across the building. While its initial performance varied, over extended periods, its performance converged with that of the *Centralized Node Deployment* algorithm. Interestingly, as the lifetime of the system increased, the performance gap between the two algorithms diminished. This finding indicates that both algorithms, despite their differing initial strategies, are well-suited for long-term communication optimization in smart buildings. Overall, our study contributes valuable insights into the strategic placement of communication nodes in smart buildings, aiming to facilitate seamless and efficient communication in the face of physical barriers. We believe that our research will serve as a strong foundation for future work in this area, potentially leading to even more efficient algorithms and strategies for communication in smart buildings. Moreover, we plan to expand smart complex infrastructure consisting of multiple number of thin walls and thick walls, as well as to apply realistic experimental environments based on the proposed framework and strategies.

## Figures and Tables

**Table 1:** Notations.

Notations	Descriptions
*S*	a 3D smart building surveillance space
*M*	a set of system members
*W*	a set of wall-recognition surveillance security barriers
δ	the number of system members
*r*	the detection range of system member
*t*	the allowed number of connections through the wall
*p*	the possible number of connections among system members
*q*	the requested number of wall-recognition surveillance security barriers
*i*	an identifier of a system member, where $i\leq \delta ,m_{i}\in M$
*j*	an identifier of a system member, where $j\leq \delta ,m_{j}\in M$
*k*	an identifier of a wall-recognition barrier, where $k\leq q,w_{k}\in W$

**Table 2:** Pros and sons of previous studies and our scheme.

Studies	Pros	Cons
[23]	- Initial work of barriers	- 2D environment
	- Sleep-wakeup scheduling	- Not practical product
	- Homogeneous capability	- Biased theoretical analysis
	- Heterogeneous capability	- Not expanded environment
[25]	- Controllable trajectories	- 2D environment
	- Static and mobile sensors	- Not practical product
	- Bidding mechanism	- Biased theoretical analysis
	- Deterministic countermeasures	- Not expanded environment
[33]	- Two-way-enabled barriers	- 2D environment
	- Slab dividing strategy	- Not practical product
	- Perpendicular detection	- Biased simulation analysis
	- Horizontal detection	- Not expanded environment
Our scheme	- 3D environment	- Sole thin wall
	- Smart building with thin wall	- Not practical product
	- Green property	- Biased simulation analysis
	- Deployment strategy with wall	- Not expanded environment

## Data Availability

The data presented in this study are available on request from the corresponding author.

## Abbreviations

The following abbreviations are used in this manuscript:

IoT	Internet of Things
UAVs	Unmanned Aerial Vehicles
WalRecogSurv	wall-recognition surveillance security barriers in smart buildings
